# Supplementary material for: Occupational exposure to Brucella spp.: A systematic review and meta-analysis
Source: PLoS Negl Trop Dis. 2020 May 11;14(5):e0008164. doi: 10.1371/journal.pntd.0008164 (PMC7252629; doi:10.1371/journal.pntd.0008164)
Supplement: S2 Appendix — (DOCX) [file pntd.0008164.s002.docx]

## S2 Appendix: Extensive overview of search terms

CABI:

| (veterinar* OR laboratorist* OR farmer* OR abattoir* OR slaughter* OR vaccinator* OR cowboy* OR student* OR butcher*) AND (expos*) AND (occupation* OR job-relat* OR professional* OR work*) AND (brucel* OR "malta fever" OR "Gibraltar fever" OR "bang’s disease") |
| --- |

Cochrane:

| (veterinar* OR laboratorist* OR farmer* OR abattoir* OR slaughter* OR vaccinator* OR cowboy* OR student* OR butcher*) AND (expos*) AND (occupation* OR job-relat* OR professional* OR work*) AND (brucel* OR "malta fever" OR "Gibraltar fever" OR "bang’s disease") |
| --- |

Pubmed:

| ((((veterinar* OR laboratorist* OR farmer* OR abattoir* OR slaughter* OR vaccinator* OR cowboy* OR student* OR butcher*)) AND expos*) AND (occupation* OR job-relat* OR professional* OR work*)) AND (brucel* OR “malta fever” OR “Gibraltar fever” OR “bang’s disease”) |
| --- |

Science Direct:

| (veterinar* OR laboratorist* OR farmer* OR abattoir* OR slaughter* OR vaccinator* OR cowboy* OR student* OR butcher*) AND (expos*) AND (occupation* OR job-relat* OR professional* OR work*) AND (brucel* OR "malta fever" OR "Gibraltar fever" OR "bang’s disease") |
| --- |

Scielo:

| (veterinar* OR laboratorist* OR farmer* OR abattoir* OR slaughter* OR vaccinator* OR cowboy* OR student* OR butcher*) AND (expos*) AND (occupation* OR job-relat* OR professional* OR work*) AND (brucel* OR "malta fever" OR "Gibraltar fever" OR "bang’s disease") |
| --- |

Scopus:

| ALL ( veterinar* OR. laboratorist* OR farmer* OR abattoir* OR slaughter* OR vaccinator* OR cowboy* OR student* OR butcher* ) AND ALL ( expos* ) AND ALL ( occupation* OR job-relat* OR professional* OR work* ) AND ALL (brucel* OR malta AND fever OR gibraltar AND fever OR bangs AND disease) |
| --- |

Web of Science:

| TS=((veterinar* OR laboratorist* OR farmer* OR abattoir* OR slaughter* OR vaccinator* OR cowboy* OR student* OR butcher*)AND(expos*)AND(occupation* OR job-relat* OR professional* OR work*)AND(brucel* OR malta fever OR Gibraltar fever OR bangs disease)) |
| --- |
