# Supplementary material for: Occupational exposure to Brucella spp.: A systematic review and meta-analysis
Source: PLoS Negl Trop Dis. 2020 May 11;14(5):e0008164. doi: 10.1371/journal.pntd.0008164 (PMC7252629; doi:10.1371/journal.pntd.0008164)
Supplement: S3 Appendix — (DOCX) [file pntd.0008164.s003.docx]

## S3 Appendix: Inclusion and exclusion criteria for selection of articles

| Inclusion criteria | Exclusion criteria |
| --- | --- |
| - All countries - All years - *Brucella* spp. - Occupational exposure to brucellosis - Studies in English, Spanish, French or Portuguese | - Epidemiological data about animals - Diagnosis of infection in animals - Genetics - Microbiology - Immunology - Molecular biology - Diagnostic performance of tests - Vaccination - Therapeutics - Full text not available |
