# Supplementary material for: Occupational exposure to Brucella spp.: A systematic review and meta-analysis
Source: PLoS Negl Trop Dis. 2020 May 11;14(5):e0008164. doi: 10.1371/journal.pntd.0008164 (PMC7252629; doi:10.1371/journal.pntd.0008164)
Supplement: S4 Appendix — (DOCX) [file pntd.0008164.s004.docx]

## S4 Appendix: Studies describing occupational human infection by *Brucella* spp.

| **First author** | **Country** | **Time period** | **Positive/**  **Tested** | **Study design** | **Diagnostic** | ***Brucella* species** | **Predictors of transmission**  **(number of individuals in each category)** |
| --- | --- | --- | --- | --- | --- | --- | --- |
| Akhvlediani | Georgia | 1970 – 1973  1988 – 1889  2004 – 2008 | 300/300 | Case series | **Criterion:** Wright and Huddelson agglutination tests, combined with clinical signs.  **Cut off:** titer ≥ 200 or in Wright and Huddelson agglutination tests. | Not reported | **Occupation:** among the cases, 87 (29.0%) were shepherds and 37 (12.3%) farmers.  **Animal contact:** 259 (86.3%) of all the patients, 121 (40.4%) with sheep and 105 (35.0%) with both sheep and cattle.  **Vaccine contact:** 1 (0.5%) patient reported exposure. |
| Al Dahouk | Germany | 1962 – 2005 | 6269/6269 | Case series | **Criterion:** positive culture or STAT or CFT or ELISA, combined with the occurrence of an acute febrile illness or two other clinical signs.  **Cut off:** only one significant titer (not described), or an increase in the titer in the follow-up serum sample. | *B. abortus*  *B. melitensis*  *B. suis* | **Occupation:** among the 102 cases in which the probable source of infection could be identified, 7 (6.9%) were laboratory technicians, 4 (3.9%) shepherds, 2 (2.0%) farmers, 4 (3.9%) butchers and 1 (1.0%) veterinary.  **Animal contact:** 16 (15.7%) direct contact with cattle, 24 (23.5%) with sheep and 16 (15.7%) with goats. |
| Al-Aska | Saudi Arabia | Not reported | 4/4 | Case report | **Criterion:** positive culture or saline agglutination method combined with clinical signs.  **Cut off:** not described, although the minimum titer reported was 280 for saline agglutination method. | *B. melitensis* | **Occupation:** all 4 (100.0%) cases were among laboratory workers.  **Positive culture contact:** 1 (25.0%) by splash contaminated solution on the face, 2 (50.0%) by contaminated aerosols and 1 (25.0%) by needlestick injury, from a needle containing contaminated synovial fluid |
| Al-Shamahy | Yemen | 1992 – 1993 | 235/469 | Case-control | **Criterion:** positive culture or STAT combined with clinical signs.  **Cut off:** titer ≥ 160 in STAT. | *B. melitensis* | **Occupation:** among the 235 cases, 90 (38.3%) were farmers, 14 (6.0%) shepherds, 14 (6.0%) laboratory workers and 3 (1.3%) abattoir workers. |
| Ari | Kenya | 2005 | 9/12 | Case report | **Criterion:** positive RBT or CFT or STAT or *Brucella* microagglutination test or rapid ELISA.  **Cut off:** titer ≥160 in STAT and *Brucella* microagglutination test, and ≥ 320 for rapid ELISA. | Not reported | **Occupation:** among the tested individuals, 12 (100.0%) were pastoralists, of which 2 (16.7%) tested positive for RBT and 7 (58.3%) for CFT. From 10 pastoralists tested by rapid ELISA and *Brucella* microagglutination test, 8 (80.0%) and 5 (50.0%) were positive, respectively. |
| Arlett | England | 1995 | 1/1 | Case report | **Criterion:** positive direct agglutination and IgM and IgG ELISA, and dye test for *Brucella*.  **Cut off:** not reported. | *B. melitensis* | **Occupation:** the 1 (100.0%) case was a veterinary microbiologist, who contracted the condition while working on the products of conception from animals. |
| Ashford | United States of America | 1998 – 1999 | 26/26 | Case series | **Criterion:** clinical signs and self-report exposure, with or without positive culture.  **Cut off:** not available. | *B. abortus* – RB51 strain | **Occupation:** among the cases, 21 (80.8%) were veterinarians, 2 (7.7%) veterinary students, 2 (7.7%) veterinary technicians and 1 (3.8%) ranch employee.  **Vaccine contact:** 21 (80.1%) had needlestick injuries, 4 (15.4%) vaccine spray on the conjunctiva and 4 (3.8%) vaccine spray on broken skin. |
| Asiimwe | Uganda | 2013 | 45/90 | Case-control | **Criterion:** positive STAT.  **Cut off:** titers ≥ 160 in STAT. | Not reported | **Occupation:** among the cases, 5 (11.1%) were farmers and 40 (88.9%) agropastoralists.  **Animal contact:** 19 (42.2%) rear goats and sheep. |
| Avdikou | Greece | 2002 – 2004 | 152/152 | Case series | **Criterion:** positive STAT, or RBT or ELISA or positive  blood culture, and/or clinical signs and/or clinician’s decision for anti-brucellosis treatment.  **Cut off:** titers ≥ 320 in STAT. | Not reported | **Occupation:** among the cases, 92 (60.5%) were shepherds, 2 (1.3%) veterinarians or assistants, 4 (2.6%) abattoir workers and 1 (0.6%) tannery worker.  **Animal contact:** 114 (75.0%) had direct contact with animals.  **Vaccine contact:** 41 (25.0%) reported contact with the REV-1 vaccine.  **Animal fluids contact:** 36.8% (56) had contact with an aborted fetus. |
| Aworh | Nigeria | 2010 – 2011 | 54/224 | Sectional | **Criterion:** positive RBT or ELISA.  **Cut off:** not reported. | *B. abortus* and *B. melitensis* | **Occupation:** among the 54 cases, 32 (59.2%) were butchers, 11 (20.4%) meat sellers and 8 (14.8%) abattoir cleaners.  **Animal fluids contact:** among the 54 cases, 29 (53.7%) reported handling aborted fetus, 35 (64.8%) slaughtering animals and 34 (63.0%) slaughtering animals with an injury.  **Personal protective equipment:** among the 54 cases, 52 (96.3%) of them reported not wearing gloves. |
| Blasco and Díaz | Spain | Not reported | 2/2 | Case report | **Criterion:** positive culture or STAT or Coombs’ test or RBT.  **Cut off:** not described, although the minimum titer reported was 320 in STAT and 1280 in Coombs’ test. | *B. melitensis* – REV-1 strain | **Occupation:** all 2 (100.0%) cases were veterinarians.  **Vaccine contact:** 2 (100.0%) had a previous contact with REV-1 vaccine and 1 (50.0%) had an accidental self-inoculation. |
| Bosilkovski | Macedonia | 1989 – 1990 2000 – 2001  2011 – 2014 | 340/340 | Case series | **Criterion:** clinical findings and detection of specific antibodies at significant titers or demonstration of a fourfold rise in antibody titer in samples obtained 3–4 weeks apart (RBT, STAT, Coombs’ test or immunocapture assay)  **Cut off:** titer ≥ 160 in STAT, ≥ 320 in Coombs’ test and ≥ 640 for immunocapture assay. | Not reported | **Occupation:** among the cases, 22 (6.5%) were veterinarians or veterinary technician.  **Animal contact:** 217 (63.8%) had reported contact with animals. |
| Bourne | Canada | 1963 | 3/3 | Case report | **Criterion:** positive culture or STAT.  **Cut off:** titers ≥ 320 in STAT | *B. abortus* | **Occupation:** all 3 (100.0%) cases were employed by or around a packing house; being 1 (33.3%) a truck driver, 1 (33.3%) work constructor and 1 (33.3%) slaughter-house employee.  **Animal fluids contact:** 1 (33.3%) handled discarded carcasses of animals, 1 (33.3%) worked as a construction worker 20 feet from a pile of animal carcasses, in a packing plant, and 1 (33.3%) worked as a slaughter-house employee. |
| Campbell | Vietnam | 2016 – 2017 | 10/10 | Case report | **Criterion:** positive culture  **Cut off:** not applicable. | *B. melitensis* | **Occupation:** among the cases, 1 (10.0%) was veterinarian.  **Animal contact:** 10 (100.0%) reported exposure to goats prior to the febrile episodes; 8 (80.0%) kept goats and 2 (20.0%) had consumed goat meat.  **Vaccine contact:** 1 (10.0%) vaccinated goats. |
| Cash-Goldwasser | Tanzania | 2012 – 2014 | 50/562 | Cohort | **Criterion:** positive culture or *Brucella* microagglutination test, combined with clinical signs.  **Cut off:** a fourfold or higher rise in *Brucella* antibody titer, between acute and convalescent serum samples. Probable cases were defined as a single titer ≥ 160 in *Brucella* microagglutination test. | Not reported | **Occupation:** among the 50 cases, 15 (30.0%) were farmers and 1 (2.0%) livestock attendant.  **Animal contact:** 3 (6.0%) assisted livestock abortions, 4 (8.0%) assisted livestock births, 9 (18.0%) cleaned livestock waste and 7 (14.0%) slaughtered livestock. |
| CDC | United States of America | 2007 – 2008 | 3/3 | Case report | **Criterion:** positive culture and PCR combined, or *Brucella* microagglutination test with clinical sign.  **Cut off:** titers ≥ 640 in *Brucella* microagglutination test. | *B. suis* | **Occupation:** all 3 (100.0%) cases were among hunters.  **Animal fluids contact:** 1 (33.3%) hand cutting with knife while field dressing one of the hunted feral swine.  **Personal protective equipment:** 2 (66.6%) of the 3 hunters adopted no individual protection measure during the handling of feral swine. |
| Čekanac | Serbia | 1980 – 2008 | 1521/1521 | Case series | **Criterion:** positive STAT, ELISA, CFT,  Coombs’ test, or fluorescent antibody test, combined with clinical signs.  **Cut off:** not reported. | Not reported | **Occupation:** among 70 infected patients from 1999 to 2006, 3 (4.3%) were laboratory workers.  **Animal contact:** among 47 outbreaks, from 1991 to 2008, 14 (30.0%) reported contact with infected animals. Among 70 infected patients from 1999 to 2006, 35 (50.0%) had direct or indirect contact with infected sheep, 3 (4.3%) with infected goats and 11 (15.7%) with other infected animals.  **Positive culture contact:** among 70 infected patients from 1999 to 2006, 3 (4.3%) reported contact with specimens from infected animals and 1 (1.4%) was accidentally exposed during specimen collection. |
| Compés Dea | Spain | 2014 | 1/1 | Case report | **Criterion:** positive culture and PCR, combined with clinical signs.  **Cut off:** not applicable. | *Brucella suis* – biovar 1 strain 1330 | **Occupation:** the 1 (100.0%) case worked in a medical waste treatment plant.  **Contact with sharp object:** 1 (100.0%) had foot poked with a needle that was lying on the floor.  **Personal protective equipment:** although properly used (boots), was not enough to prevent infection. |
| Cooper | Saudi Arabia | 1988 | 150/300 | Case-control | **Criterion:** positive culture or STAT, and 2-ME or Coombs’ test, all combined with clinical signs.  **Cut off:** titers ≥ 1280 in STAT, 2-ME or Coomb’s test. | Not reported | **Occupation:** among the 150 cases, 85 (56.6%) were animal owners.  **Animal contact:** among the 150 cases, 111 (74.0%) had direct contact with animals; 1 (0.6%) cows, 12 (8.0%) camels, 63 (42.0%) sheep and 35 (23.3%) goats. Among 150 cases, 58 (38.6%) slaughtered animals and 47 (31.3%) helped animal parturition. |
| Dean | Togo | 2011 | 7/683 | Sectional | **Criterion:** positive in RBT or ELISA.  **Cut off:** not reported | Not reported | **Occupation:** among the 7 cases, 6 (85.7%) were from pastoralists communities. |
| Demirdal | Turkey | Not reported | 3/3 | Case report | **Criterion:** positive culture or RBT, STAT or biochemical reactions, combined with clinical signs.  **Cut off:** not described, although the minimum titer reported was 1:640 for STA. | *B. melitensis* | **Occupation:** all 3 (100.0%) cases were among laboratory workers.  **Positive culture contact:** no accident occurred. Although using masks, transmission was probably due to aerosol contamination because of the current practice of sniffing culture plates. 3 (100.0%) of the professionals were found to be working on the specimen of the index case patient. |
| Ergonul | Turkey | 2000 - 2003 | 12/12 & 7/55 | Case-control | **Criterion:** clinical illness combined with positive blood culture or STAT.  **Cut off:** titer ≥ 320 in STAT. | *B. melitensis* – biovar 1 and 3 | **Occupation:** all 12 (100.0%) cases were among healthcare workers; 6 (50.0%) were physicians, 1 (8.0%) was a laboratory technician, 1 (8.0%) was a nurse, 2 (17.0%) were secretaries and 2 (17.0%) were staff disposing contaminated laboratory material.  **Probable reason of infection:** 7 (58.0%) were processing cultures, 2 (16.0%) were disposing of laboratory material and 3 (26.0%) were eating and drinking near the microbiology bench.  **Risk factor analysis (multivariate analysis):** male physicians had a higher risk of *Brucella* infection (P = 0.008), and using gloves was found to be protective (P = 0.017). |
| Fiori | Italy | 1990 – 1991 | 12/12 | Case report | **Criterion:** positive RBT, microagglutination test or STAT.  **Cut off:** not described, although the minimum titer reported was 320 in STAT. | *B. abortus* | **Occupation:** all 12 (100.0%) cases were among laboratory workers.  **Positive culture contact:** 12 (100.0%) of cases worked in the same laboratory where the outbreak occurred. It originated from the accidental rupture of a polystyrene centrifuge tube containing *B. abortus* biotype 1 atypical strain (previously isolated from a camel). |
| Gelfand | United States of America | Not reported | 1/1 | Case report | **Criterion:** positive culture and serology for *Brucella* (not specified) combined with clinical signs.  **Cut off:** not described, although the minimum titer reported was 160 for IgM and 1280 for IgG. | *B. suis* | **Occupation:** the 1 (100.0%) case was hunter.  **Animal fluids contact:** 1 (100.0%) hunted, field-dressed, and butchered wild pigs several weeks before the onset of illness. |
| Gonçalves | Brazil | 2007 | 3/207 | Sectional | **Criterion:** positive in RBT and positive in  2-ME.  **Cut off:** not described, although the minimum titer reported was 50 in 2-ME. | Not reported | **Occupation:** all 3 (100.0%) cases were among rural residents and animal owner.  **Animal fluids contact:** 2 (66.6%) had performed artificial insemination. |
| Gruner | Switzerland | 1990 – 1991 | 5/5 | Case report | **Criterion:** positive culture or *Brucella* microagglutination test, IgM and IgG ELISA or CFT combined or not with clinical signs.  **Cut off:** not described, although the minimum titer reported was 160 in *Brucella* microagglutination test. | *B. melitensis* | **Occupation:** all 5 (100.0%) cases were among laboratory workers.  **Positive culture contact:** 2 (40.0%) had worked with *Brucella* strains (isolated from index case I); 3 (60.0%) had worked with *Brucella* strains (isolated from index case II).  **Personal protective equipment:** all the  index cases were handled without any specific safety precautions, such as work under a safety hood, wearing of gloves, masks and/or protective glasses. |
| Guney | Turkey | 2007 | 1/1 | Case report | **Criterion:** positive cultured and *Brucella* agglutination test combined with clinical signs.  **Cut off:** not described, although the minimum titer reported was 320 in *Brucella* agglutination test. | *B. melitensis* | **Occupation:** the 1 (100.0%) case was farmer. |
| Hartady | Malaysia | 2013 | 1/1 | Case report | **Criterion:** positive PCR, RBT and STAT combined with clinical sings.  **Cut off:** not described, although the minimum titer reported was 40 in STAT. | *B. melitensis* | **Occupation:** the 1 (100.0%) case was laboratory worker.  **Positive culture contact:** 1 (100.0%) were involved in the isolation of *B. melitensis* from goats, for the past 3 months. |
| Hasanjani Roushan | Iran | 1997 – 2003 | 469/469 | Case series | **Criterion:** positive STAT or 2-ME combined with clinical signs.  **Cut off:** titers ≥ 320 in STAT and ≥ 160 in 2-ME. | Not reported | **Occupation:** among the cases, 53 (11.3%) were animals breeders, 7 (1.5%) veterinarians and 38 (8.1%) laboratory workers. |
| Hendricks | United States of America | 1959 – 1960 | 128/1627 | Case series | **Criterion:** positive culture or blood agglutination test combined or not with clinical signs.  **Cut off:** not described, although the minimum titer reported was 160 for blood agglutination test. | *B. suis* and *B. melitensis* | **Occupation:** all 128 (100.0%) cases were among employees of a swine-slaughtering plant.  **Animal fluids contact:** according to labor department, the cases were: 60 (33.7%) of 178 from killing, 24 (12.2%) of 197 from cutting, 20 (24.4%) of 82 from casing, 4 (7.4%) of 54 from head trimming, 3 (5.3%) of 57 from the freezer, 3 (9.4%) of 32 from inedible, 3 (4.3%) of 69 from the maintenance room, 2 (4.4%) of 45 from the pork plant, 2 (4.3%) of 46 from curing, 1 (1.9%) of 54 from boning, 1 (2.2%) of 45 from night cleaning-up, 0 (0.0%) of 450 from other departments, 3 (1.0%) of 300 from management and office, 2 (18.2%) of 11 from killing inspection and 0 (0.0%) of 7 from other inspection sectors. |
| Jia | China | 2001 – 2005 | 10/10 | Case report | **Criterion:** positive culture or STAT combined with clinical signs.  **Cut off:** titers ≥ 100 in STAT. | *B.*  *melitensis* | **Occupation:** among the cases, 5 (50.0%) farmers, 3 (30.0%) herdsmen, 1 (10.0%) teacher and 1 (10.0%) student who lived in rural area.  **Animal contact:** 7 (70.0%) had a history of close contact with cattle and sheep. |
| Joffe | United States of America | Not reported | 1/1 | Case report | **Criterion:** positive culture and *Brucella* agglutination test (not specified) combined with clinical signs.  **Cut off:** not described, although the minimum titer reported was 80. | *B. abortus* – S19 strain | **Occupation:** the case 1 (100.0%) was veterinary.  **Vaccine contact:** 1 (100.0%) accidentally injected the *B. abortus* – S19 vaccine strain into the skin, between the hand palm and the right thumb. |
| Kiel | Saudi Arabia | 1983 – 1990 | 9/9 | Case report | **Criterion:** positive culture or *Brucella* serology combined with clinical sings.  **Cut off:** not described, although the minimum titer reported was 1280. | *B. melitensis* | **Occupation:** all 9 (100.0%) cases were hospital employees; 7 (77.8%) bacteriology technologists, 2 (22.2%) nurses and 1 (11.1%) obstetrician.  **Positive culture contact:** 1 (11.1%) recalled sniffing next to bacteriology plates, as part of his diagnostic approach, and 1 (11.1%) removed culture plates from the safety hood, for closer visual examination.  **Positive patient assistance:** 1 (11.1%) disclosed that occasionally participated in precipitous deliveries when wearing gloves was not possible; 1 (11.1%) probably had contact with the body fluids of one of the 76 brucellosis patients hospitalized. |
| Kozukeev | Kyrgyzstan | 2003 | 100/200 | Case-control | **Criterion:** positive Wright agglutination test combined with clinical signs.  **Cut off:** titers > 100 in Wright agglutination test. | Not reported | **Occupation:** among the 100 cases, 12 (12.0%) were farm workers and 86 (86.0%) reported owning animals at home, of which 80 (80.0%) were cattle, 70 (70.0%) goats, 50 (50.0%) dogs and 49 (49.0%) sheep.  **Animal contact:** among 86 cases that reported having farm animals at home, 70 (81.3%) cleaned barns, 60 (69.7%) assisted in animal delivery and 22 (25.6%) slaughtered animals. |
| Mailles | France | 2004 – 2013 | 250/250 | Case series | **Criterion:** positive culture or PCR or a four-fold or greater rise in *Brucella*  antibody titers between acute and convalescent phase (RBT, Wright test, competitive ELISA, Brucellacapt lateral flow immunochromatography and indirect immunofluorescence for IgM and IgG detection).  **Cut off:** not reported. | *B. suis* and *B. melitensis* | **Occupation:** among the 250 cases, 213 (85.2%) were imported, of which 13 (6.1%) reported occupational risk while staying in the endemic country, being 11 (5.2%) cattle breeders, 1 (0.5%) veterinarian and 1 (0.5%) agronomist; among domestic cases 37 (14.8%), 17 (45.9%) involved laboratory workers.  **Animal contact:** among the 213 imported cases, 113 (53.1%) had direct contact with animals that could be a possible source of *Brucella*, being 45 (21.1%) with sheep, 38 (17.8%) with goats and 30 (14.1%) with cows; among 37 domestic cases, 4 (10.8%) had never traveled out of the country, but lived in cattle farms before the country was declared officially free of brucellosis, and 1 (2.7%) reported an episode of brucellosis in the herd. |
| Mailles | France | 2004 – 2016 | 7/7 | Case series | **Criterion:** clinical  signs consistent with brucellosis and (1) for a confirmed case, the isolation of a *Brucella* spp.  strain from any clinical sample,  (2) for a probable case: a fourfold or greater increase in *Brucella* antibody titers between acute and convalescent phase serum specimens obtained at least  3–4 weeks apart, or the detection of *Brucella* DNA in a clinic specimen by PCR and (3) for a possible case: a single elevated serum  *Brucella* total antibody titer.  **Cut off:** not reported. | *B. suis* | **Occupation: a**mong the 7 cases, 6 (85.7%) were  wild boar hunters and occupationally infected. 1 (14.3%) patient was not a hunter, she only prepared and cooked a piece of  a wild boar carcass.  **Animal fluids contact:** **a**mong the 7 cases, 6 (85.7%) with probable repeated and massive exposure to *Brucella suis* biovar 2 as they dressed (namely skinned and gutted) wild boars after hunting.  **Personal protective equipment:** **a**mong the 7 cases, 6 (85.7%) dressed wild boars after hunting without any individual protection. |
| Mamani | Iran | 2014 – 2015 | 29/218 | Sectional | **Criterion:** positive STAT retested with Coombs’ and Wright test, and negative STAT retested with 2-ME.  **Cut off:** titers ≥ 80 in Wright test. | Not reported | **Occupation:** among the 29 cases, 19 (16.9%) occurred among 112 butchers, 7 (8.1%) among 86 slaughterhouse workers and 3 (15.0%) among 20 veterinarians.  **Personal protective equipment:** among 112 butchers, 72 (64.3%) used masks, 16 (14.3%) goggles, 5 (4.5%) gloves, 38 (33.9%) boots and 48 (42.9%) apron. Among 86 slaughterhouse workers, 10 (11.6%) used masks, 4 (4.7%) goggles, 20 (23.3%) gloves, 75 (87.2%) boots and 53 (61.6%) apron. Among 20 veterinarians, 12 (60.0%) used masks, 7 (35.0%) goggles, 12 (60.0%) gloves, 6 (30.0%) boots and 9 (45.0%) apron. |
| Martin-Mazuelos | Spain | 1998 | 4/4 | Case report | **Criterion:** positive culture and RBT, along with Coombs’ and microagglutination test, all combined with clinical signs.  **Cut off:** not described, although the minimum titer reported was 40. | *B. melitensis* | **Occupation:** all 4 (100.0%) cases were among laboratory workers.  **Positive culture contact:** 4 (100.0%) handled blood cultures. No accident occurred in the laboratory and the blood cultures were reported to be handle correctly, except that a biosafety hood was not used. |
| Memish | Saudi Arabia | 1991 – 2000 | 7/7 | Case report | **Criterion:** positive culture or STAT combined with clinical signs.  **Cut off:** titers ≥ 320 in STAT. | *B. melitensis* and *B. abortus* | **Occupation:** all 7 (100.0%) cases were among laboratory workers.  **Positive culture contact:** 6 (85.7%) worked with positive *Brucella* cultures and 1 (14.3%) sniffed and handled, outside of the biosafety cabinet, a culture of *Brucella* erroneously identified as gram positive; 1 (14.3%) visited a laboratory where *Brucella* cultures were tested. |
| Mousa | Kuwait | 1984 – 1985 | 379/379 | Case series | **Criterion:** positive culture or STAT or immunofluorescence test.  **Cut off:** titer ≥ 160 in STAT and ≥ 640 in immunofluorescent or a four-fold rise in titers. | *B. melitensis* | **Occupation:** among the cases, 151 (39.8%) were students, 92 (24.3%) housewives, 29 (7.7%) retired, 24 (6.3%) shepherds, 29 (7.7%) unemployed, 21 (5.5%) soldiers, 17 (4.5%) civil servants, 8 (2.1%) skilled workers, 5 (1.3%) traders and 2 (0.5%) farmers and 1 (0.3%) engineer.  **Animal contact:** 42 (11.1%) reported direct contact with animals and denied consuming raw milk. |
| Mufinda | Angola | 2012 | 39/323 | Sectional | **Criterion:** positive STAT or RBT.  **Cut off:** titers ≥ 160 in STAT combined with positive result in RBT. | Not reported | **Occupation:** among the 39 cases, 7 (17.9%) of 131 were slaughterhouse workers and 32 (82.1%) of 192 were cattle breeders.  **Animal contact:** among the 32 cattle breeders, 25 (78.1%) reported that abortion remains were abandoned in the pasture and eventually ingested by dogs and pigs |
| Nicoletti | United States of America | 1984 | 1/1 | Case report | **Criterion:** positive STA combined with clinical sings.  **Cut off:** not described, although it was reported an eight-folder titer to 1:160 a month, after the accident. | *B. abortus* – S19 strain | **Occupation:** the 1 (100.0%) case was veterinary student.  **Vaccine contact:** 1 (100.0%) accidentally injected himself during vaccination of a calf. |
| Noviello | United States of America | 2001 – 2002 | 2/2 | Case report | **Criterion:** positive culture and STAT  **Cut off:** not described, although the minimum titer reported was 640 in STAT. | *B. melitensis* | **Occupation:** all 2 (100.0%) cases were laboratory workers.  **Positive culture contact:** approximately two months before illness, laboratory worker 2 had personally processed laboratory worker 1’s blood culture, but had characterized the isolate as coryneform bacilli, in a class II biosafety cabinet. |
| Ozaras | Turkey | Not reported | 1/1 | Case report | **Criterion:** positive culture and Wright test combined with clinical signs.  **Cut off:** not described, although the minimum titer reported was 1280 for Wright test. | Not reported | **Occupation:** the 1 (100.0%) case was laboratory worker. |
| Pisani | Uruguay | 2009 – 2010 | 14/14 | Case series | **Criterion:** positive RBT and ELISA IgM combined with clinical signs.  **Cut off:** not described, although 85.7% (12) reported titers of 320 for ELISA IgM. | Not reported | **Occupation:** all 14 (100.0%) cases were among slaughtered workers.  **Animal fluids contact:** 8 (57.1%) of the cases worked at slaughter department, 1 (7.1%) at viscera sector, 1 (7.1%) at cattle shed, 2 (14.3%) cleaning, 1 (7.1%) at maintenance room and 1 (7.1%) at tripe room.  **Personal protective equipment:** 0 (0.0%) used goggles. |
| Proch | India | 2015 - 2016 | 64/279 | Sectional | **Criterion:** positive  RBT, or STAT and IgG ELISA.  **Cut off:** titer ≥ 80 in STAT, and optical density between 0.456 and 0.798 for IgG ELISA. | Not reported | **Occupation:** all 64 (100.0%) cases were among veterinarians.  **Vaccine contact:** among 296 interviews, 12 (4.1%) reported a *Brucella* needlestick injury.  **Animal contact:** among the 296 individuals interviewed about the activities done a month before the survey, 205 (69.3%) had attended parturitions, 106 (35.8%) handled aborted fetus, 191 (64.5%) handled retained placenta, 132 (44.6%) handled stillbirth, 222 (75.0%) performed artificial insemination and 216 (73.0%) attended infertility cases.  **Personal protective equipment:** among the 296 individuals interviewed about the protection measures adopted a month before the survey, 130 (44.7%) did not use it while handling healthy animals, 82 (28.1%) did not use it while handling sick animals, 245 (84.8%) did not use it in parturition, 265 (92.7%) did not use it while handling feces and urine, 257 (88.3%) did not use it while handling aborted fetus and stillbirth, and 256 (87.7%) did not use it while handling retained placenta. |
| Rodrigues | Brazil | 2012 | 3/11 | Case report | **Criterion:** positive IgM or IgG ELISA combined or not with clinical signs.  **Cut off:** not reported. | *B. abortus* | **Occupation:** all 3 (100.0%) cases were among laboratory workers.  **Positive culture contact:** 3 (100.0%) were inside the laboratory, were media seeded with *B. abortus* and manipulated in a damage biological safety cabinet.  **Personal protective equipment:** 3 (100.0%) reported wearing laboratory coats, disposable gloves and N95 masks regularly, when they were inside the laboratory. |
| Rodríguez Valín | Spain | 1998 – 1999 | 28/106 | Case-control | **Criterion:** positive culture or RBT, along with STAT and Coombs’ test, combined with clinical signs.  **Cut off:** titers ≥ 80 in STAT and ≥ 320 in Coomb’s test. | *B. melitensis* | **Occupation:** among the cases, 14 (50.0%) worked at slaughtered department, 17.9% (5) at residues sector, 12 (7.14%) at chamber, 1 (3.6%) at cleaning, 1 (7.14%) at maintenance room, 3 (10.7% ) at external companies and 1 (3.6%) at sanity services.  **Animal fluids contact:** 13 (46.4%) of the 28 cases reported cutting themselves with dirty sharps.  **Personal protective equipment:** among the 106 cases, 9 (32.1%) did not use gloves, 10 (35.7%) did not use mask and 12 (42.9%) did not use goggles. |
| Sam | Malaysa | 2009 | 4/51 | Case report | **Criterion:** positive STAT, RBT, Coombs’ test and CFT.  **Cut off:** titers ≥ 80 in STAT. | *B. melitensis* | **Occupation:** all 4 (100%) cases were among laboratory workers.  **Positive culture contact:** blood cultures were handled openly on the bench; the class II biosafety cabinet was not used and there was no directional airflow system in the laboratory. |
| Smith | United States of America | 1979 | 1/1 | Case report | **Criterion:** positive culture and STAT combined with clinical signs.  **Cut off:** not described, although the minimum titer reported was 1280 in STAT. | *B. melitensis* | **Occupation:** the 1 (100.0%) case was laboratory worker.  **Positive culture contact:** work with positive blood cultures on an open bench. |
| Sofian | Iran | 2005 | 150/300 | Case-control | **Criterion:** positive STAT and 2-ME combined with clinical signs.  **Cut off:** titers ≥160 in STAT combined titers ≥ 20 in 2-ME. | Not reported | **Occupation:** among the cases, 50 (33.3%) were farmers, 1 (0.7%) butcher, 8 (5.3%) animal husbandry and 4 (2.7%) farmers and animal husbandry.  **Animal contact:** among the cases, 133 (88.7%) raised cattle, and 102/133 (76.7%) had vaccinated their animals, although no question of accidental exposure was studied. |
| Staszkiewicz | United States of America | 1988 | 8/75 | Case report | **Criterion:** positive culture or STAT combined with clinical signs.  **Cut off:** titers ≥ 160 in STAT. | *B. melitensis* biovar 3 | **Occupation:** all 8 (100.0%) cases were among laboratory workers.  **Positive culture contact:** 6 weeks before the first case of brucellosis occurred, a positive isolate was handled on an open workbench and not in a biologic safety cabinet. The original patient isolate and all employee isolates were identified at the Centers for Disease Control as *B. melitensis* biovar 3. |
| Strbac | Serbia | 2000 – 2014 | 102/102 | Case series | **Criterion:** STAT, Wright or *Brucella* microagglutination test.  **Cut off:** not reported. | Not reported | **Occupation:** among the cases, 23 (22.5%) were agriculturists, 12 (11.8%) were rearer, 11 (10.8%) were veterinarians, 7 (6.9%) were cattlemen and 3 (2.9%) were stockbreeders.  **Animal contact:** 44 (43.1%) cases indicated daily direct contact with domestic animals. |
| Tee | England | 1969 | 1/1 | Case report | **Criterion:** positive blood culture and saline agglutination test, along with CFT and 2-ME, all combined with clinical signs.  **Cut off:** not described, although the minimum titer reported was 320 inr saline agglutination, less than 5 in CFT and 640 in 2-ME. | *B. abortus* | **Occupation:** the 1 (100.0%) case was farmer. |
| Thomas | England | 1991 – 1996 | 3/404 | Cohort | **Criterion:** positive CFT and microagglutination test, or IgG and IgM ELISA.  **Cut off:** titers ≥ 80 in CFT, ≥ 20 in microagglutination test and ≥ 80 in IgG and IgM ELISA. | Not reported | **Occupation:** all 3 (100.0%) cases were farmers. |
| Tsegay | Ethiopia | 2013 – 2014 | 2/149 tested | Sectional | **Criterion:** RBT as a screening test and CFT as confirmatory  **Cut off:** not reported | Not reported | **Occupation:** among the cases, CFT confirmed 1 (0.7%) loader and 1 (0.7%) cleaner with positive diagnosis.  **Animal fluids contact:** 76 (48.7%) of 156 interviewed workers related accidentally cut during slaughtering and eviscerating.  **Personal protective equipment:** among the 156 respondents, 127 (81.4%) replied not using gloves and 135 (86.6%) reported not covering their mouth during slaughtering and eviscerating process. |
| Wallach | Argentina | 1999 – 2006 | 21/30 | Case series | **Criterion:** positive for at least two tests STAT, 2-ME, RBT or CFT, combined or not with clinical signs.  **Cut off:** titers ≥ 100 in STAT. | Not confirmed, but probably *B. abortus* – S19 strain | **Occupation:** all 21 (100.0%) cases were among workers from S19-manufacturing plants.  **Vaccine contact:** among all employees, 9 (30.0%) related some kind of exposure; 6 (20.0%) percutaneous, 3 (10.0%) inhalatory  and 1 (3.3%) conjunctival (more than one route of exposure). |
| Wallach | Argentina | Not reported | 1/1 | Case report | **Criterion:** positive culture combined with clinical signs. | *B. canis –* M strain | **Occupation:** the 1 (100.0%) case was laboratory worker (production of *B. canis –* M-strain used for serologic diagnosis).  **Positive culture contact:** 1 (100.0%) handled a positive culture outside of a biological safety cabinet and related resuspension, by repeated pipetting with his mouth.  **Personal protective equipment:** 1 (100.0%) did not use any protection. |
| Wallach | Argentina | 2014 – 2015 | 17/17 | Case series | **Criterion:** positive culture or RBT confirmed by STAT, CFT and competitive ELISA, all combined with clinical signs.  **Cut off:** titers ≥ 100 in STAT, 10 in CFT or 28.0% of inhibition percentage in competitive ELISA. | *B. suis* | **Occupation:** RBT and STAT were positive for 17 (100.0%) patients. CFT and competitive ELISA were performed in 14 cases and were positive in 13 (92.9%) of them. Blood cultures were positive in 14 (82.3%) of the 17 patients. Among the cases, 17 (100.0%) were workers from a pork processing plant. Information about work was available for 11 patients, of which 7 (63.6%) worked in the killing area and 4 (43.64%) worked at animal cleaning and transportation.  **Personal protective equipment:** 17 (100.0%) used goggles, gloves and masks. |
| Wallach | Argentina | Not reported | 33/60 | Case series | **Criterion:** positive culture or RBT, along with STAT, 2-ME, and IgG and IgM ELISA.  **Cut off:** titers ≥ 50 in STAT and 2-ME; values of absorbance > 30 for ELISA. | *B. melitensis* | **Occupation:** among the cases, 25 (75.8%) were rural workers.  **Animal contact:** among 23 individuals who reported continuous contact with goats, 21 (91.3%) developed brucellosis, and only 11 (32.0%) of 28 individuals who reported occasional contact became ill. Only 1 (11.0%) of 9 individuals who reported no contact with goats developed brucellosis. |
| Williams | England | 1968 – 1969 | 30/30 | Case report | **Criterion:** positive STAT, 2-ME, antihuman globulin test and CFT, all combined with clinical signs.  **Cut off:** not described, although the minimum titer reported was 640 in STAT, 320 in CFT, 5120 in antihuman globulin test and 1280 in 2-ME. | Not reported | **Occupation:** among the cases, 21 (70.0%) were farmers or farmworkers. |
| Yagupsky | Israel | 1997 | 7/7 | Case report | **Criterion:** positive culture or positive RBT confirmed with STAT and 2-ME, combined or not with clinical signs.  **Cut off:** titers ≥ 160 in STAT and ≥ 20 in 2-ME. | *B. melitensis* | **Occupation:** all 7 (100.0%) cases were among hospital personnel, who worked or visited a clinical microbiology laboratory.  **Positive culture contact:** a *Brucella* culture was misidentified as *Streptococcus* and manipulated outside the safety cabinet. |
| Zhan | China | 2005 | 3/14 | Case report | **Criterion:** positive saline agglutination test combined with clinical signs.  **Cut off:** not described, although the minimum titer reported was 800 for saline agglutination test. | Not reported | **Occupation:** all 3 (100.0%) cases were processing sheep placenta.  **Animal fluids contact:** 3 (100.0%) processed sheep placenta and reported had already splashed animal fluids on face.  **Personal protective equipment:** 3 (100.0%) used gauze mask and rubber glove, but did not wash their hands with disinfectant after work. |

Tests used: RBT = Rose bengal test; STAT = Standard tube agglutination test; CFT = Complement fixation test; ELISA = Enzyme-linked immunosorbent assay; 2-ME = 2-Mercaptoetanol
