# Supplementary material for: Occupational exposure to Brucella spp.: A systematic review and meta-analysis
Source: PLoS Negl Trop Dis. 2020 May 11;14(5):e0008164. doi: 10.1371/journal.pntd.0008164 (PMC7252629; doi:10.1371/journal.pntd.0008164)
Supplement: S5 Appendix — (DOCX) [file pntd.0008164.s005.docx]

## S5 Appendix: Number of *Brucella* species isolated per worker category

|  | Rural workers | Abbatoir workers | Veterinarians | Laboratory workers | Hunters | Total |
| --- | --- | --- | --- | --- | --- | --- |
| *B. melitensis* | 23 | 12 | 3 | 33 |  | 71 |
| *B. suis* |  | 21 |  |  | 9 | 30 |
| *B. abortus* | 4 | 1 | 2 | 3 |  | 10 |
| *B. canis* |  |  |  | 1 |  | 1 |
| Total | 27 | 34 | 5 | 37 | 3 | 112 |
