# Supplementary material for: Occupational exposure to Brucella spp.: A systematic review and meta-analysis
Source: PLoS Negl Trop Dis. 2020 May 11;14(5):e0008164. doi: 10.1371/journal.pntd.0008164 (PMC7252629; doi:10.1371/journal.pntd.0008164)
Supplement: Attachment — Submitted filename: Revision Note PNTD.pdf [file pntd.0008164.s007.pdf]

UNIVERSIDADE FEDERAL DE LAVRAS
PROGRAMA DE PÓS-GRADUAÇÃO
EM CIÊNCIAS VETERINÁRIAS
Caixa Postal: 3037 CEP: 37200-900 Lavras – MG
Phone: (35) 38291735

Lavras, February 11th, 2020.

PNTD-D-19-01642

Dear Dr. Tao Lin

Associate Editor
PLOS Neglected Tropical Diseases

Dear Dr. Elsio Wunder Jr

Deputy Editor
PLOS Neglected Tropical Diseases

Thank you for your message regarding our manuscript “*Occupational exposure to human brucellosis infection: a systematic review and meta-analysis*”, PNTD-D-19-01642.

We would like to thank you for your kindness and interest in considering our manuscript. Furthermore, we would like to thank the reviewers for their appropriate and useful suggestions regarding our manuscript. All the points made by reviewers are consistent and improved the manuscript.

Please find the enclosed **Responses to reviewers**.

Sincerely yours,

Profa. Elaine Maria Seles Dorneles
Laboratório de Epidemiologia Molecular e Biologia Celular
Departamento de Medicina Veterinária
Universidade Federal de Lavras, UFLA

UNIVERSIDADE FEDERAL DE LAVRAS
PROGRAMA DE PÓS-GRADUAÇÃO
EM CIÊNCIAS VETERINÁRIAS
Caixa Postal: 3037 CEP: 37200-900 Lavras – MG
Phone: (35) 38291735

Responses to reviewers

- **REVIEWER#1**

Editorial and data presentation modifications:

Include S4 Appendix in the manuscript.

Answer: We inserted the S4 appendix in the manuscript as figure 1 as requested.

General comments:

In order to add new knowledge through the review, the following points should be taken in consideration in a revised manuscript: for 1538 cases, there was only 106 isolation of Brucella spp, i.e. less than 7%. Therefore, all the discussion related to the importance of Brucella species per worker category should be introduced by a word of caution.

Answer: The discussion related to the importance of each *Brucella* species were corrected by adding the number of species identified compared with the total number of brucellosis cases reported for each worker category, as can be seen below:

○ Rural workers: "Rural workers are among the group most affected by brucellosis, mainly caused *B. melitensis* observed in the present study, totaling 27 individuals with direct diagnosis of isolation and identification of the *Brucella* species, among the 870 cases observed in this group (Fig 3a)."

○ Abbatoir workers: "The second group most affected by occupational brucellosis (n = 292), mainly by *B. suis* (n = 21), followed by *B. melitensis* (n = 12) and *B. abortus* (n = 1) (Fig 3a), were butchers and abattoir workers, probably due to the regular manipulation of sharp objects and to close contact with potentially infected animals and their organs."

○ Veterinarians and veterinary assistants: "In fact, the accidental exposure to brucellosis vaccines has great significance to brucellosis cases among veterinarians and assistants (n = 189), being confirmed by direct diagnostic methods that revealed *Brucella* spp. infection caused by *B. melitensis* (n = 3) and *B. abortus* (n = 2) vaccine strains (2, 50, 52, 53)."

○ Laboratory workers: "Interestingly, this group (n = 183) showed the greatest number of species isolated: *B. melitensis* (n = 33), *B. abortus* (n = 3) and *B. canis* (n = 1) (Fig 3a), which could be explained by the wide variety of clinical specimens that are often handled by those professionals in the diagnostic routine."
○ Hunters: "The occupation with the lowest number of infected individuals identified was the group of hunters (n = 10), which differently from the previous groups exhibited exclusively *B. suis* isolates (n = 9) (Fig 3a)."

Specify per worker category the number of Brucella isolation.
Answer: We provided the complete information in the S5 Appendix: number of *Brucella* species isolated per worker category.

A specific section devoted to human brucellosis due to RB51 should be included, although the vast majority of such cases are linked to the consumption of raw milk.
Answer: As the focus of our review was the occupational character of brucellosis, the importance of RB51 exposure was highlighted among veterinarians and assistants, related to unintended exposure to vaccines. However, the importance of RB51 accidental exposure was discussed, as can be seen: "Accidental exposures to brucellosis live attenuated vaccines are especially important when they occur with RB51, since antibodies against this strain are not detected by routine serological tests and RB51 is resistant to rifampicin, one of the preferential drugs to treat human brucellosis (90)."

Why is B. canis not discussed in this review although it appears in figure 2?
Answer: The importance of isolation of *B. canis* from a laboratory worker was included in the discussion for this occupational group as recommended: "The isolation of *B. canis* in a worker in this occupational group is noteworthy, as it was caused by the M- strain, a strain used for the serologic diagnosis of canine brucellosis that has reduced virulence in dogs (75)."

Laboratory workers: specify the importance of this group in countries where brucellosis has been eradicated. In addition, it would be worth to mention that mis diagnosis (as Ochrobactrum antropi) occurs regularly.

Answer: The importance of this occupational group in European countries (where brucellosis has been eradicated in some countries), as well as the mis diagnosis by physicians and the misidentification of the organism were discussed in the following sentence: "*Brucella* spp. cultures must only be handled in laboratories with biosafety level 3 or higher (92); however, due the lack of specificity of the clinical signs caused by the disease, associated with the effectiveness of public policies in some European countries (19, 31), where brucellosis occurs primarily among travelers, many physicians rarely raise the hypothesis of brucellosis when sending biological samples for laboratory analysis, leading to exposure to the agent during manipulation of the clinical material by the microbiologist (93). Misidentification of the organism also happens and puts the health of individuals who manipulate cultures at risk (63, 67)."

Consider including S4 Appendix in the manuscript.

Answer: We inserted the S4 appendix in the manuscript as figure 1, as requested.

Although the systematic review is proficiently executed, the information is to a large extent not new. Therefore, the authors should discuss in dept which new information has been generated through their work. In this perspective, the meta-analysis is perhaps the most interesting part of this research.

Answer: The authors recognize that the innovations achieved with systematic review and meta-analysis have not been properly valued and discussed. We appreciate the suggestion and to overcome this deficiency we added the following sentences highlighting the new information and scientific contribution provided by the present study: "The lack of accurate information on the quantification and peculiarities of the risk of brucellosis in each occupational group makes it difficult to direct public resources for the control and prevention of brucellosis in individuals most likely to present the disease, especially in a context with several other demands which also require a portion of the available funds, already limited. In this context, this systematic review provided a meticulous understanding of the risk factors peculiar to each of the main occupations (farmers, slaughterhouses, veterinarians, laboratories and hunters) closely related to *Brucella* spp. infection. Our results also revealed the great lack of information from these occupational groups on the importance of applying preventive measures to minimize the risk of transmission

of brucellosis during work. In addition, through meta-analysis it was possible not only to confirm the occupational character of brucellosis, widely recognized, but also to quantify this risk in an unprecedented way in the scientific literature through the calculation of odds ratios, systematically compiling studies so far dispersed in the literature."

Specific comments:

Title: not Ok, I suggest: Occupational exposure to Brucella spp.: a systematic review and meta-analysis

Answer: We modified the tittle as suggested: "Occupational exposure to *Brucella* spp.: a systematic review and meta-analysis".

L27: live attenuated anti-brucellosis vaccines

Answer: We corrected the term as suggested.

L41: different wildlife species

Answer: We corrected the term as recommended.

L45: live attenuated anti-brucellosis vacines

Answer: We corrected the term as suggested.

L69-72: introduce the DALY concept

Answer: The DALY concept was inserted and exemplified with a study that performs its calculation for human brucellosis in India in 2018, both for occupational groups and for the adult population not occupationally exposed to *Brucella* spp.: "Damage caused by the disease in individuals' quality of life is intangible and the economic losses attributed to the infection in humans are associated to the costs of hospital treatment, drugs and absence from work due to disabling feature of the disease in its severe form (6). These damages are more intense in groups frequently exposed to microorganisms of the genus *Brucella*: the Disability-Adjusted Life Year (DALY), a metric that quantifies the burden of mortality and morbidity caused by a disease, were found to be 0.13 [95% uncertainty interval (UI) 0.06 - 0.18] per thousand persons per year

in non-occupational adult and 0.29 [95% UI; 0.08 - 0.70] per thousand persons per year in occupational population (farmers, abattoir workers and veterinarians) for human brucellosis in India (10), in which one DALY can be thought of as one lost year of "healthy" life."

L76: live attenuated anti-brucellosis vaccines

Answer: We corrected the term as suggested.

L167-169: specify to which animal species the aborted fetus belongs

Answer: We specify that abortion materials were from production animals: "Moreover, another study also identified that aborted fetus remains from production animals were abandoned in the pasture and eventually ingested by dogs and pigs, in some properties in Angola (33)."

L189: live attenuated anti-brucellosis vaccines

Answer: We corrected the term as suggested.

L222: reports on contamination by B. suis biovar 2 in France should be mentioned in this section

Answer: The authors included the article requested in the study and made the necessary changes in the figures, supplementary materials and in the text: "Job-related exposure was described in hunters in three papers, totalizing 10 infected individuals, from America and Europe (77-79). Contact with animal fluid was reported, and in France, *B. suis* biovar 2 was isolated from six hunters, all of whom reported not using any type of personal protective equipment while eviscerating the carcasses of slaughtered animals (79)."

L258-264: the explanation is imported cases of brucellosis in non-endemic countries, not scientific interest

Answer: The total number of individuals affected in each country has no relation to the total number of cases of human brucellosis in that particular location, as well as whether they are autochthonous or allochthonous. USA, the country with the largest number of studies on brucellosis (nine), is not necessarily the one with the biggest occurrence of this disease in its population (indeed only 0.4 annual cases per million of population). Instead, Turkey, despite

the higher incidence of the disease in its territory (262.2 annual cases per million of population) has a smaller number of high quality scientific publications (three) (Pappas et al., 2006). Thus, the number of studies and cases of human brucellosis is much more related to the demand and interest of researchers on this subject than the impact that this disease has on each nation. Reference: Pappas G, Papadimitriou P, Akritidis N, Christou L, Tsianos EV. The new global map of human brucellosis. The Lancet Infectious Diseases. 2006;6(2):91-9. This statement is the discussion section.

L271: B. abortus is the most important species isolated in ref 86. However, other references, particularly from China show that B. melitensis is the most important species, whereas in Kazakhstan, B. melitensis is exclusively isolated from human patients

Answer: We corrected this paragraph: "Rural workers are among the group most affected by brucellosis, mainly caused *B. melitensis*, totaling 27 individuals with direct diagnosis of isolation and identification of the *Brucella* species, among the 870 cases observed in this group (Fig 3a)."

L272-274: the symptoms are for all Brucella species. It is thus incorrect to ascribe them to B. abortus

Answer: As the previous sentence citing *B. abortus* was removed, the sentence about the symptoms associated with this species was also deleted: "Rural workers are among the group most affected by brucellosis, mainly caused *B. melitensis*, totaling 27 individuals with direct diagnosis of isolation and identification of the *Brucella* species, among the 870 cases observed in this group (Fig 3a)."

L343: all systematic review should follow the PRISMA statement

Answer: The authors thank the statement, however we kept this sentence in the study as the information that the PRISMA methodology is a strength and we add the comment recognizing that this methodology is recommended in all systematic review studies: "The greatest strengths of this paper are that it is based on the PRISMA statement (as recommended for conducting systematic reviews)".

274: Brucella melitensis in not more pathogenic than B. abortus in humans. It is just the most prevalent species!

Answer: We have added information about the higher prevalence of this species in humans: "These results are especially important to public health, since *B. melitensis* is one of the most virulent and the most prevalent species of *Brucella* spp. for humans (87)." However, Young (1995) highlighted in table 2 that *B. melitensis*, together with *B. suis* biovar 5, are more virulent for humans, compared with *B. abortus* and with others biovars of *B. suis*, characterized by moderate virulence. Reference: Young EJ. An overview of human brucellosis. Clinical infectious diseases: an official publication of the Infectious Diseases Society of America. 1995;21(2):283-9; quiz 90.

L281: reference

Answer: We added the reference as recommended. "The close contact of rural workers with small ruminants, preferred hosts for *B. melitensis*, was identified as the main form of acquisition of the disease among these individuals (Table 1), which has been confirmed by the identification of a high genetic similarity between *B. melitensis* strains isolated from occupationally infected workers and from goat milk samples (38)."

L304: laboratory worker: see general comment

Answer: We corrected as suggested.

L325: hunters: for which countries is this group important?

Answer: The authors pointed out that such groups were found in two countries where hunting is cultural: "The occupation with the lowest number of infected individuals identified was the group of hunters (n = 10), which differently from the previous groups exhibited exclusively *B. suis* isolates (n = 9) (Fig 3a). Hunting, a widespread activity in United States of America and in some European countries, such as France, is often associated with the primary route of transmission for *B. suis* in humans: through the contact and dressing of carcasses (5, 90). Therefore, the presence of bacteria in the muscular tissues of boars is sufficient to cause infection in humans, especially when carried out without the proper use of individual protection measures."

UNIVERSIDADE FEDERAL DE LAVRAS
PROGRAMA DE PÓS-GRADUAÇÃO
EM CIÊNCIAS VETERINÁRIAS
Caixa Postal: 3037 CEP: 37200-900 Lavras – MG
Phone: (35) 38291735

L293: it should be highighted her and in the abstract that veterinarians are the groups for which exposure to vaccine strains is the most importance

Answer: We correct the sentence as recommended in the discussion: "Subsequently, veterinarians and veterinary assistants comprised the third occupational group most affected by brucellosis. In addition to contact with secretions and excretions of potentially infected animals, activities inherent to their work (56), these individuals are the ones with the most important exposure to *Brucella* spp. live attenuated vaccines (REV-1, S19 and RB51) (Table 3), which are a source of the infection for humans (2)." Due to the limitation of 300 characters in the abstract, it was not possible to include such information.

L326-328: this is a wrong statement. B. suis infection is mainly due to contact and dressing of carcasses, not consumption of meat

Answer: We correct the information as suggested: "The primary route of transmission for B. suis in general population is usually through the contact and dressing of carcasses (5, 89). Therefore, the presence of bacteria in the muscular tissues of boars is sufficient to cause infection in humans, especially when carried out without the proper use of individual protection measures."

L340: wording: expressive?

Answer: We replace "expressive" for "considerable": "However, it is important to take into account that despite the low number of studies used in the meta-analysis, the total number of individuals analyzed (n = 1069) and those with occupational brucellosis (n = 269) was considerable, supporting the robust results observed (Fig 4)."

L350: L354: The One Health concept comes out of the blue and should either be deleted or better explained

Answer: The One Health concept was explained and contextualized in the following paragraph: "These data on human cases of occupational brucellosis can be used as a first step towards adopting a One Health approach, which is an interdisciplinary collaboration that aims to reduce the occurrence of zoonotic diseases in humans, through the prevention of such diseases in

animals (95). Thus, the control of brucellosis could be conducted more efficiently and strategically, in order to reduce the incidence of the disease not only in humans, but also in animals and the environment.”

Figure 2: this figure may give a wrong impression because the status towards brucellosis has changed (several countries became "officially free" from brucellosis) during the study period. For example, for Germany, Canada, USA abattoir workers are mentioned although this is not true anymore. In addition, an explanation must be given on why only veterinarians in India and only abattoir workers in Nigeria are reported while many rural worker are reported in other African countries. Answers to this type of questions would possibly generate new information.
Answer: The number shown in the figure has no relation to the country's status in relation to human brucellosis. It only portrays the number of cases attributed to occupational exposure in each group of workers by country and continent. A search was carried out at WAHIS in order to check the status of each country for brucellosis and then mark it with a symbol in figure 3 to differentiate the free countries from the countries that still have the disease. However, there are countries that submitted reports in 2019, countries that submitted last in 2018 and countries that never submitted reports. There are still very variable conditions to be presented in the figure: some territories are free of *B. abortus* in domestic and wild animals, others are free only in domestic animals. There is also a variation regarding to brucellosis situation, as some countries are free for *B. melitensis* but are not free for *B. suis* or *B. abortus*. Such extremely diverse and changeable characteristics among countries make it impossible to provide disease status information in the image content. Thus, to improve the reader's understanding, the time interval (when available) in which the studies were carried out in each country was provided in figure 3 a, as well as it had already been made available in supplementary material S4, leaving no room for doubt. In addition, this discussion section highlights the information that studies identified in the systematic review, not necessarily are related to the status of brucellosis in the country: "However, it is very important to mention that the number of infected individuals and the number of papers published by country do not have a direct relationship with the current prevalence of occupational brucellosis in that locality, but is more related to scientific interests of local researchers In fact, USA showed the biggest number of studies published, although it is one of the countries with the lowest incidence of human brucellosis in the world (1). "

- **REVIWER#2**

Summary:

This publication performed an analysis (meta-) of the frequency of *Brucella* infection associated with certain occupations and the need to address this failure by providing personal protection (PPE) to perform work with potentially contaminated products. The results match previous publications demonstrating increased risk associated with specific activities. In general, increased interaction with infected animals or the products of infected animals mirrored increased levels of human and animal infection. The potential for aerosolisation during some procedures is another factor associated with increased risk. As has been made clear over the last 100 years working around or with animals susceptible to *Brucella* infection increases the risk of human infection.

Strengths:

The authors have exhaustively screened the literature (seven databases) for publications related to Brucella infection identifiable or distinguishable by occupation and confirmed using bacteriological and molecular approaches to confirm direct linkages between human activity/profession, and animal infection. Toward this goal, the authors have exhaustively searched the literature for related publications and used a duplicative screening method to enhance accuracy. In general, the manuscript is well-written with exceptions described below.
Answer: The authors acknowledge the comments about the manuscript.

Weaknesses:

The manuscript provided is a review or mini-review, but is laid out in a form reminiscent of a primary publication with appropriate sections (Introduction, Methods, Results, Discussion and Conclusion). As a result, there is an elevated level of duplication of text which could be

UNIVERSIDADE FEDERAL DE LAVRAS
PROGRAMA DE PÓS-GRADUAÇÃO
EM CIÊNCIAS VETERINÁRIAS
Caixa Postal: 3037 CEP: 37200-900 Lavras – MG
Phone: (35) 38291735

eliminated to provide better focus and reduce the size of the document, especially the Discussion section.

Answer: Although conventional reviews present the text continuously, divided into sections related to the subject, according to the PRISMA methodology, a meta-analysis should contain all sections of a standard article. Thus, in the results section, the information that returned in the systematic search was presented, while the discussion section is based on comments on the meaning of the results, the comparison with other research findings and the author's position on the subject. The entire text was strategically organized in order to first present the results of each occupational group from the most exposed to the least exposed, as well as the discussion, in order to avoid going back and forth in the same information repeatedly.

The bottom line is that the meta-analysis, which is a novel feature of the manuscript, is only briefly described. Overall the study provides little added insight relative to the human/animal cycle of infection, including only 6 lines of results in a manuscript exceeding 360 lines of text. The results are by no means a surprise, since the level of occupational exposure/infection parallel the previous literature with or without a meta-analysis. The finding of a 3.5-fold increase in potential for acquiring disease for workers exposed to the agent over workers who are not potentially exposed provides little real-world usefulness other than to predict an increased likelihood that flu-like symptoms are more likely to be brucellosis related. However, the authors might consider if such an approach could be used to improve training.

Answer: The authors appreciate the comment. Despite being a very relevant information for the study, the exposure of the results of the meta-analysis is quite simple, since it is a calculation of an odds ratio. However, this was not the only result related to the cycle of human and animal brucellosis infection. In this systematic review it was possible to identify each of the most recurrent risk factors for each occupational group most exposed: although they are all grouped as "most likely to come into contact with the pathogen", each of them has its particularities, such as the manipulation of uterine secretions from infected animals, neglects the use of personal protective equipment, accidental exposure to live attenuated vaccines, errors in the identification and manipulation of microorganism cultures and habits of eviscerating carcasses in environments with unfavorable conditions. Thus, the great relevance of the present study is not only for compiling such information, but also for quantifying the risk that these individuals

are more likely to develop the disease when compared to individuals not exposed to work. The authors are also grateful because this reviewer's comment caused a perception that the importance of the work described above may not have been properly explored in the manuscript. Thus, in order to provide a better understanding and practical application of this study for readers, we added the following paragraphs related to the discussion of meta-analysis and the relevance of the study not only for the scientific environment but for its benefit to the community: "The occupational character of human brucellosis is supported by the results generated from the meta-analysis of 3 case-control studies, which showed that animal breeders, laboratory workers and abattoir workers were significant more likely to become infected with Brucella spp. strains than people who develop other job-related activities (OR 3.47; 95% CI: 1.47 to 8.18) (Fig 4). The low number of selected studies with a case control design (n = 3) observed among the articles resulted in the small number of high-quality papers eligible for meta-analysis. It occurred because of the impossibility of access to data of exposed and non-exposed individuals. However, it is important to take into account that despite the low number of studies used in the meta-analysis, the total number of individuals analyzed (n = 1069) and those with occupational brucellosis (n = 269) was considerable, supporting the robust results observed (Fig 4). Those data revealed the weight of exposure to Brucella spp. during labor activities for the occurrence of human brucellosis, which is essential to take into account for the design of strategies to minimize its occurrence. (…) The lack of accurate information on the quantification and peculiarities of the risk of brucellosis in each occupational group makes it difficult to direct public resources for the control and prevention of brucellosis in individuals most likely to present the disease, especially in a context with several other demands which also require a portion of the available funds, already limited. In this context, this systematic review provided a meticulous understanding of the risk factors peculiar to each of the main occupations (farmers, slaughterhouses, veterinarians, laboratories and hunters), closely related to *Brucella* spp. infection. Our results also revealed the great lack of information from these occupational groups on the importance of applying preventive measures to minimize the risk of transmission of brucellosis during work. In addition, through meta-analysis it was possible not only to confirm the occupational character of brucellosis, widely recognized, but also to quantify this risk in an unprecedented way in the scientific literature through the calculation of odds ratios, systematically compiling studies hitherto dispersed in the literature. Since the human cases of

occupational brucellosis in this study present direct and continuous contact with animals or their fluids, responsible for the transmission of the pathogen and the development of the disease in these individuals, these data can be used as a first step towards adopting a One Health approach, which is an interdisciplinary collaboration that aims to reduce the occurrence of zoonotic diseases in humans, through the prevention of such diseases in animals (94). Thus, the control of brucellosis, which could be conducted more efficiently and strategically, in order to reduce the incidence of the disease not only in humans, but also in animals and the environment."

Specific Comments:

(lines 53-4) The report of annual incidence dates from 2006. The authors might consider the use of the approach documented to estimate or update the frequency of infection.

Answer: Unfortunately, after a wide search, as far as the authors are aware, this reference is still the most current with regard to the occurrence of human brucellosis worldwide.

The figures need to be separated from the text and provide greater detail so that they are understood without reference to the text.

Answer: The authors modified the figure legend to optimize the understanding of the figures independently of the text:

"Fig 1. PRISMA Flow diagram of selected studies.

Fig 2. Geographical and temporal distribution of the selected articles included in the present study. (a) Distribution and frequency of occupational brucellosis studies published by country (performed with aid of online dataset: https://commons.wikimedia.org/wiki/Atlas_of_the_world). (b) Distribution and frequency of occupational brucellosis studies publish by continent and decade, from 1962 to 2018.

Fig 3. Distribution of occupations affected by occupational brucellosis by country with the time interval for carrying out the studies included in this systematic review (a) and the *Brucella* species identified through direct diagnostic methods (b).

Fig 4: Forest plot of odds ratio for brucellosis among risk work groups (animal breeders, farmers, abattoir workers and laboratory workers) exposed and other individuals not

occupationally exposed to *Brucella* spp. during their labor activities. Year indicates the period in which study was performed."
