# Supplementary material for: Occupational exposure to Brucella spp.: A systematic review and meta-analysis
Source: PLoS Negl Trop Dis. 2020 May 11;14(5):e0008164. doi: 10.1371/journal.pntd.0008164 (PMC7252629; doi:10.1371/journal.pntd.0008164)
Supplement: Attachment — Submitted filename: Critique.docx [file pntd.0008164.s006.docx]

**Summary**

This publication performed an analysis (meta-) of the frequency of *Brucella* infection associated with certain occupations and the need to address this failure by providing personal protection (PPE) to perform work with potentially contaminated products. The results match previous publications demonstrating increased risk associated with specific activities. In general, increased interaction with infected animals or the products of infected animals mirrored increased levels of human and animal infection. The potential for aerosolisation during some procedures is another factor associated with increased risk. As has been made clear over the last 100 years working around or with animals susceptible to *Brucella* infection increases the risk of human infection.

**Strengths**

The authors have exhaustively screened the literature (seven databases) for publications related to *Brucella* infection identifiable or distinguishable by occupation and confirmed using bacteriological and molecular approaches to confirm direct linkages between human activity/profession, and animal infection. Toward this goal, the authors have exhaustively searched the literature for related publications and used a duplicative screening method to enhance accuracy. In general, the manuscript is well-written with exceptions described below.

**Weaknesses**

The manuscript provided is a review or mini-review, but is laid out in a form reminiscent of a primary publication with appropriate sections (Introduction, Methods, Results, Discussion and Conclusion). As a result, there is an elevated level of duplication of text which could be eliminated to provide better focus and reduce the size of the document, especially the Discussion section. The bottom line is that the meta-analysis, which is a novel feature of the manuscript, is only briefly described. Overall the study provides little added insight relative to the human/animal cycle of infection, including only 6 lines of results in a manuscript exceeding 360 lines of text. The results are by no means a surprise, since the level of occupational exposure/infection parallel the previous literature with or without a meta-analysis. The finding of a 3.5-fold increase in potential for acquiring disease for workers exposed to the agent over workers who are not potentially exposed provides little real-world usefulness other than to predict an increased likelihood that flu-like symptoms are more likely to be brucellosis related. However, the authors might consider if such an approach could be used to improve training.

**Specific Comments**

1. (lines 53-4) The report of annual incidence dates from 2006. The authors might consider the use of the approach documented to estimate or update the frequency of infection.
2. The figures need to be separated from the text and provide greater detail so that they are understood without reference to the text.
